# Primary Signet-Ring Cell Carcinoma of the Colon: A Rare and Aggressive Tumor

**DOI:** 10.7759/cureus.108307

**Published:** 2026-05-05

**Authors:** Sarra El Fassi, Hassan Ouaya, Sanae Chaib, Jinane Kharmoum, Aicha Akjay, Houda Meyiz, Mariame Chraibi, Ihsane Mellouki

**Affiliations:** 1 Department of Gastroenterology and Hepatology, Mohammed VI University Hospital, Faculty of Medicine and Pharmacy of Tangier, Abdelmalek Essaâdi University, Tangier, MAR; 2 Department of Pathology, Mohammed VI University Hospital, Faculty of Medicine and Pharmacy of Tangier, Abdelmalek Essaâdi University, Tangier, MAR

**Keywords:** case report, colon cancer, colorectal neoplasms, primary signet-ring cell carcinoma, rare tumor

## Abstract

We report the case of a 50-year-old man who presented with progressive abdominal distension, weight loss, and ascites. Imaging revealed ileocecal wall thickening associated with peritoneal involvement. Colonoscopy demonstrated a circumferential, stenosing, ulcerative lesion in the cecum that was friable and could not be traversed by the endoscope. Histopathological examination of biopsy specimens confirmed signet-ring cell carcinoma. Upper gastrointestinal endoscopy showed no evidence of a gastric primary tumor, and computed tomography excluded distant metastasis, supporting a primary colonic origin. The patient was started on XELOX chemotherapy (capecitabine and oxaliplatin). This case highlights the diagnostic challenge of this entity, particularly in the presence of atypical features.

## Introduction

Colorectal cancer ranks among the most frequently diagnosed malignancies worldwide and represents a major cause of cancer-related mortality. In Morocco, it is the second most common digestive malignancy after gastric cancer [1].

Primary signet-ring cell carcinoma (SRCC) of the colon is a rare histological subtype, accounting for approximately 0.5-2.6% of all colorectal carcinomas [2], and less than 1% of colon tumors overall [3,4]. It was first described by Laufman and Saphir in 1951 as a distinct pathological entity characterized by diffuse infiltration and aggressive behavior [5].

Although primary SRCC most frequently arises in the stomach, it may also occur in other organs, including the pancreas, breast, bladder, thyroid, and, rarely, the colon and prostate [6]. It affects both men and women and is often diagnosed at a younger age compared with conventional adenocarcinoma [7,8]. Most cases are identified at an advanced stage due to nonspecific clinical presentation and rapid tumor progression [7,8].

We report a rare case of primary SRCC of the colon in a 50-year-old patient.

## Case presentation

A 50-year-old male, former smoker (20 pack-years), with no significant past medical history and no family history of malignancy, was admitted for evaluation of progressive abdominal distension evolving over three months.

The patient reported early postprandial vomiting, unintentional weight loss of 10 kg over two months, low-grade fever, and general deterioration. There were no changes in bowel habits and no rectal bleeding.

The patient reported regular bowel movements with no history of constipation or diarrhea. There was no significant change in stool frequency, consistency, or caliber, and no mucus discharge was noted. Despite the presence of a stenosing lesion that was not traversable by endoscopy, the absence of marked bowel habit changes may be explained by partial luminal obstruction and the relatively short duration of symptoms.

On physical examination, body weight was 64 kg and height 172 cm. Vital signs were stable. The abdomen was distended with flank dullness consistent with ascites. No palpable mass was detected.

Laboratory findings are summarized in Table 1. Ascitic fluid analysis revealed high protein content (52 g/L) and an albumin level of 30 g/L. The interferon-gamma release assay was positive; however, sputum examination for acid-fast bacilli and GeneXpert Mycobacterium tuberculosis/rifampicin (MTB/RIF) were negative. No malignant cells were detected in the ascitic fluid, and microbiological studies were negative.

**Table 1:** Laboratory findings. AST: aspartate aminotransferase; ALT: alanine aminotransferase; LDH: lactate dehydrogenase; ADA: adenosine deaminase.

Parameter	Value	Reference range
Hemoglobin (g/dL)	13.7	12–16
Hematocrit (%)	40.7	36–46
Mean corpuscular volume (fL)	88.2	80–100
Mean corpuscular hemoglobin concentration (g/dL)	33.5	32–36
Platelets (×10³/µL)	298	150–400
Urea (g/L)	0.33	0.15–0.45
Creatinine (mg/L)	9.46	6–12
Aspartate aminotransferase (AST) (U/L)	28	<40
Alanine aminotransferase (ALT) (U/L)	24	<40
C-reactive protein (mg/L)	20	<5
Ascitic fluid protein (g/L)	52	<25
Lactate dehydrogenase (U/L)	164	140–280
Adenosine deaminase (U/L)	30.8	<40

**Figure 1:**
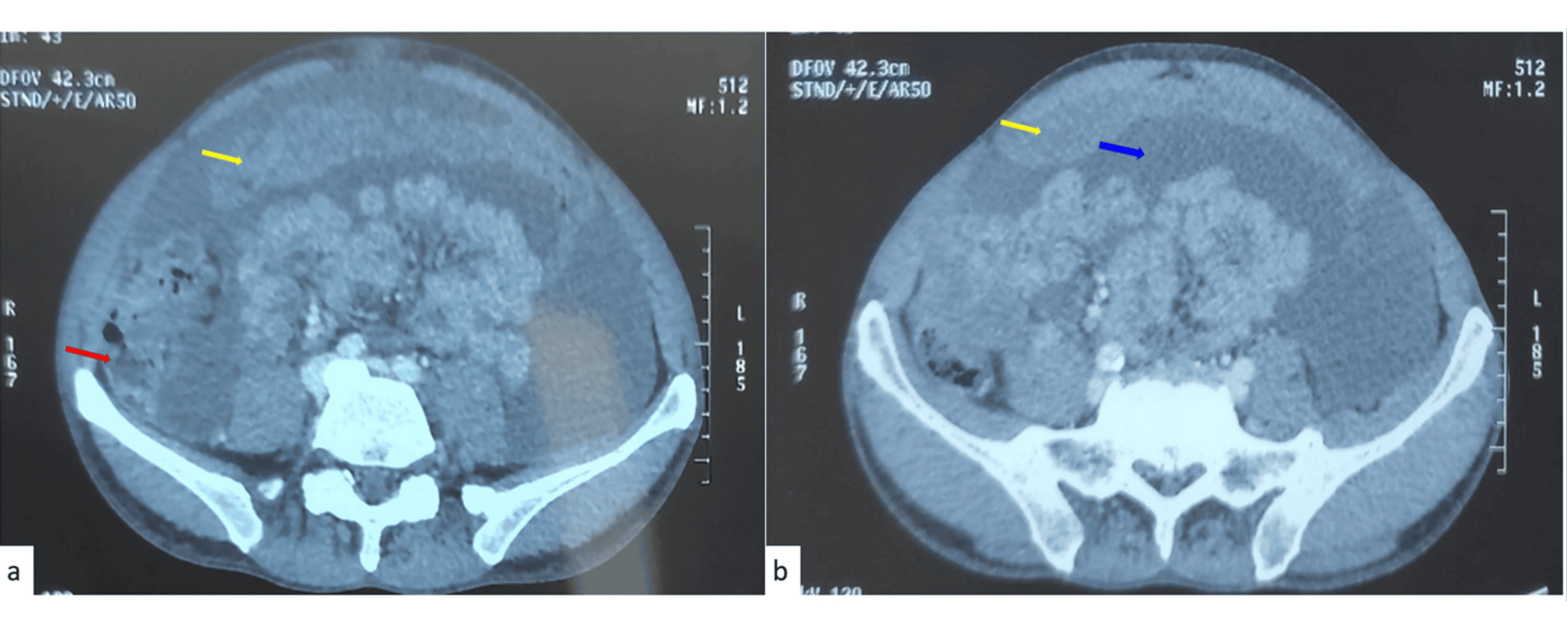
Contrast-enhanced abdominal CT scan of the abdomen and pelvis showing ileocecal wall thickening (red arrow in a), peritoneal enhancement (yellow arrow in a and b), and abundant ascites (blue arrow in b).

Abdominal ultrasound demonstrated moderate ascites. Contrast-enhanced CT scan revealed circumferential thickening of the ileocecal wall associated with omental caking and peritoneal enhancement (Figure 1), without hepatic metastasis.

**Figure 2:**
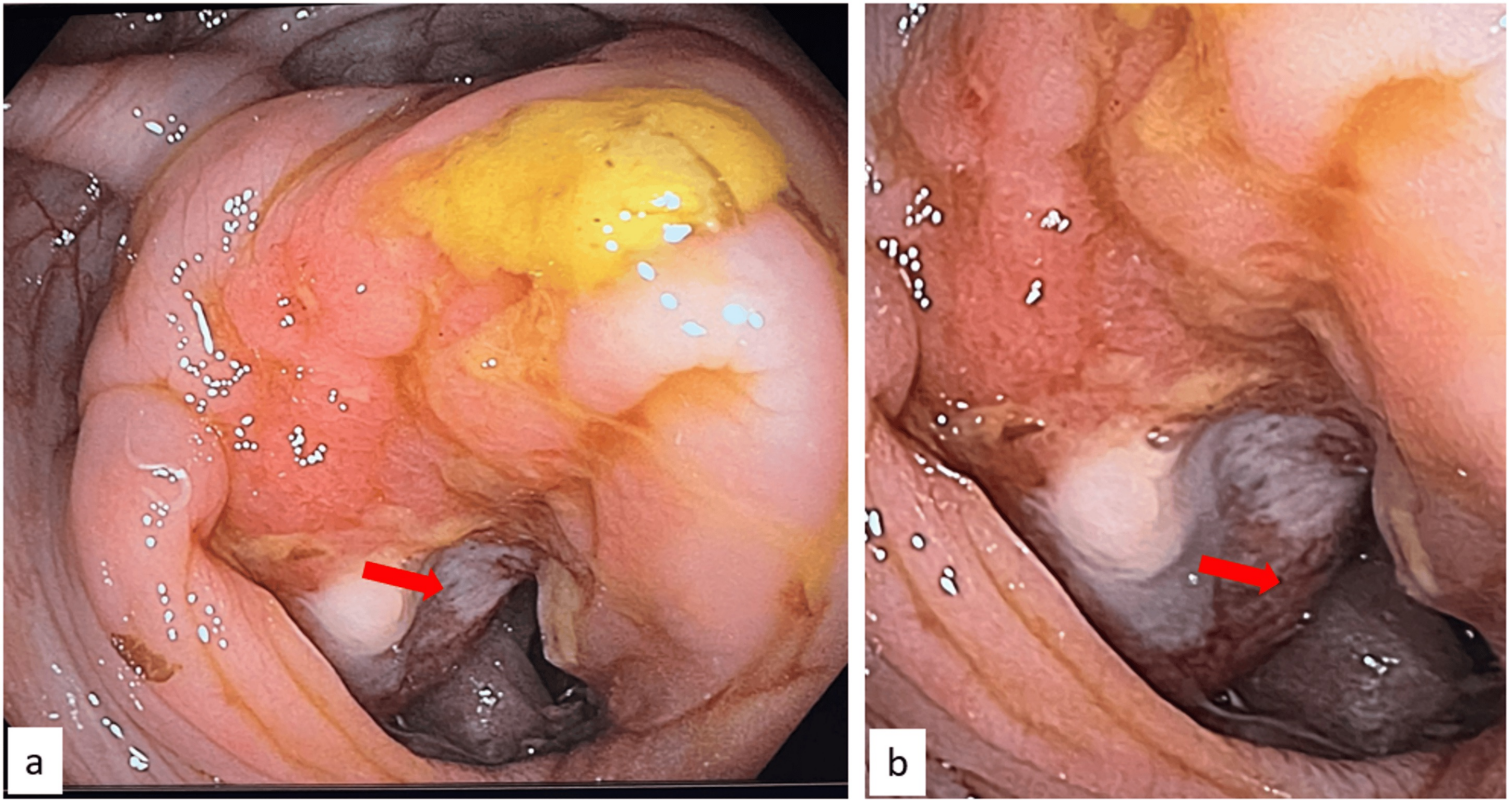
Endoscopic images. (a, b) Circumferential stenosing ulcerative lesion involving the distal cecum (red arrows).

Upper gastrointestinal endoscopy showed no evidence of gastric malignancy. Colonoscopy revealed a circumferential, ulcerative, stenosing mass in the lower cecum, friable and not traversable by the endoscope (Figure 2).

This finding may be explained by the cecal location, where the wide luminal diameter and liquid stool content allow significant narrowing without typical obstructive symptoms.

**Figure 3:**
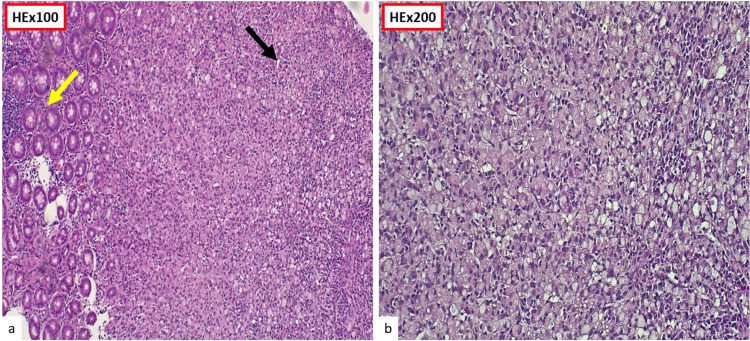
Histopathological examination. (a) Low-power view (HE ×100) showing infiltration of the colonic mucosa (yellow arrow) by poorly cohesive tumor cells (black arrow). (b) Higher magnification (HE ×200) highlighting diffuse infiltration by signet-ring cells.

**Figure 4:**
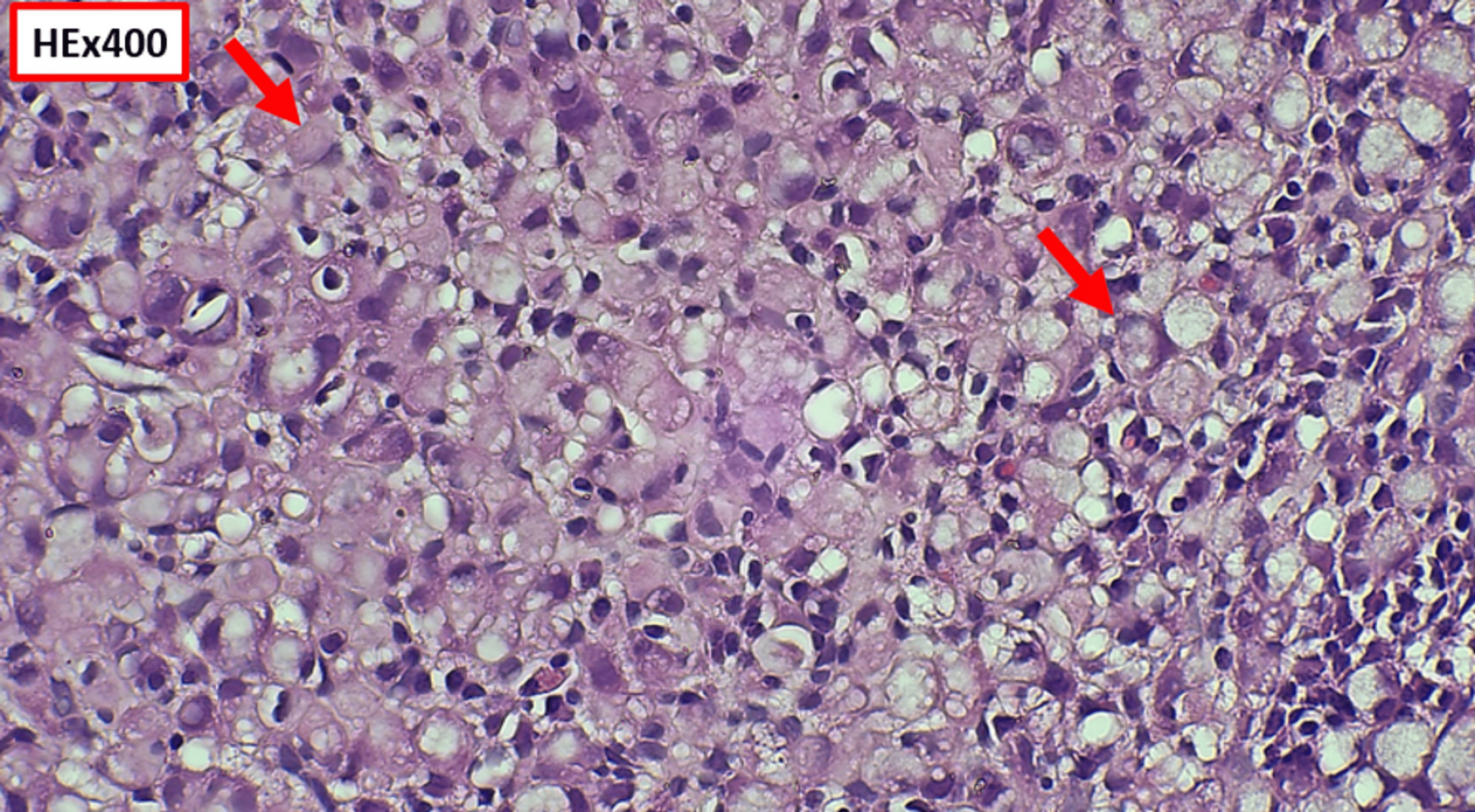
Histological section showing, at high magnification, predominantly poorly cohesive tumor cells with moderate cytonuclear atypia, hyperchromatic nuclei, and frequently vacuolated cytoplasm displacing the nucleus to the periphery, consistent with a signet-ring cell appearance (red arrow).

Histopathological examination of colonic biopsies demonstrated poorly cohesive malignant cells with intracytoplasmic mucin displacing the nucleus, consistent with SRCC (Figure 3, Figure 4). The signet-ring component represented more than 50% of tumor cells (Figure 3).

Given the absence of a gastric primary lesion on endoscopy and imaging, a diagnosis of primary SRCC was established.

The tumor was staged as advanced disease with peritoneal involvement according to the TNM classification. Given the advanced stage, the treatment intent was palliative, and systemic chemotherapy was initiated.

The patient was started on XELOX chemotherapy (capecitabine plus oxaliplatin). Therapeutic paracentesis was performed as needed.

## Discussion

Primary colorectal SRCC represents an uncommon histological subtype of colorectal cancer, with a reported incidence ranging from 0.5% to 2.6% of all colorectal malignancies [2]. It was first described by Laufman and Saphir in 1951 as a distinct pathological entity characterized by diffuse infiltration and rapid progression [5].

The reported incidence varies geographically. Western series describe rates between 0.3% and 2.6% [7,8], whereas higher proportions have been reported in certain Middle Eastern countries such as Jordan and Lebanon [9-11]. In the Moroccan series, the reported prevalence was 2.6% [11].

Clinically, SRCC commonly presents with nonspecific symptoms such as abdominal pain, changes in bowel habits, and rectal bleeding [12]. Notably, approximately 20-30% of cases have been associated with chronic ulcerative colitis, although the precise pathogenic role of inflammatory bowel disease remains unclear [12,13].

The absence of obstructive symptoms in our case, despite the presence of a stenosing lesion, can be explained by its location in the cecum, where the wide luminal diameter and liquid stool content allow significant narrowing without typical changes in bowel habits.

One of the major characteristics of SRCC is its delayed diagnosis. Approximately 78% of patients are diagnosed at advanced stages (III or IV), frequently with lymph node involvement (77%) and peritoneal dissemination (39%), while liver metastases appear less common (3%) [14]. Bonello et al. [15] proposed three main explanations for this delay: (1) the rarity of the tumor, (2) intramucosal spread with relative mucosal sparing, and (3) radiological similarity to inflammatory conditions.

In our case, the presence of ascites and peritoneal involvement initially raised suspicion of peritoneal tuberculosis, highlighting the diagnostic challenge in endemic areas.

From a molecular perspective, microsatellite instability has been reported in 33-50% of cases, whereas Kirsten rat sarcoma viral oncogene homolog mutations appear less frequent. Despite optimal therapeutic management, SRCC demonstrates low sensitivity to chemotherapy and is associated with poor survival outcomes, with reported five-year survival rates as low as 9% [14].

Regarding tumor localization, most studies report a predominance in the right colon, while approximately 20% occur in the rectum [16]. Interestingly, some authors describe a predominance in the distal colon, emphasizing the importance of tumor location in distinguishing primary colonic SRCC from metastatic gastric signet-ring carcinoma [13].

Histologically, the diagnosis requires the presence of more than 50% signet-ring cells characterized by abundant intracytoplasmic mucin displacing the nucleus, typically positive on periodic acid-Schiff or Alcian blue staining [8,16]. Differential diagnosis includes mucinous adenocarcinoma and metastatic gastric carcinoma. Unlike mucinous carcinoma, SRCC shows diffuse infiltration by poorly cohesive cells without prominent gland formation [13].

Excluding a metastatic gastric origin is mandatory before establishing the diagnosis of primary colonic SRCC. Comprehensive endoscopic and radiologic evaluation is therefore required [1]. In our patient, upper gastrointestinal endoscopy revealed no gastric lesion, supporting a primary colonic origin.

The carcinogenic pathway of colorectal SRCC remains incompletely understood. The inflammation-metaplasia-dysplasia-carcinoma sequence has been proposed, with early molecular alterations including tumor protein p53 (TP53) mutations [17].

Therapeutic management depends largely on staging and accurate pathological classification (primary versus metastatic tumor) [18]. According to the American Society of Clinical Oncology (ASCO) guidelines, adjuvant chemotherapy for stage II colon cancer is recommended only in the presence of high-risk features such as T4 stage, inadequate lymph node sampling, obstruction, perforation, poor differentiation, or lymphovascular/perineural invasion [19]. Recent studies suggest that chemotherapy efficacy in the adjuvant setting may not significantly differ between SRCC and conventional adenocarcinoma, although overall prognosis remains poorer in SRCC [16].

## Conclusions

Primary colonic SRCC is an aggressive and uncommon tumor that is often diagnosed at an advanced stage. This case highlights the diagnostic challenge posed by its nonspecific clinical presentation and the importance of thorough histopathological and radiological evaluation to exclude metastatic disease. Despite appropriate management, prognosis remains poor, underscoring the need for early recognition and multidisciplinary care.
